# A Guide to Patients with Acute Transient Vestibular Symptoms in the Emergency Department

**DOI:** 10.3390/brainsci16070754

**Published:** 2026-07-17

**Authors:** Alexander A. Tarnutzer, Arlindo C. Lima Neto, Diego Kaski

**Affiliations:** 1 Neurology, Cantonal Hospital Baden, 5404 Baden, Switzerland; 2 Faculty of Medicine, University of Zurich, 8006 Zurich, Switzerland; 3 Department of Otorhinolaryngology, University of São Paulo, São Paulo 05403-000, Brazil; aclimanetoent@gmail.com; 4 SENSE Research Unit, Department of Clinical and Movement Neurosciences, University College London, London WC1N 3BG, UK; d.kaski@ucl.ac.uk

**Keywords:** vertigo, dizziness, acute management, differential diagnosis, emergency department

## Abstract

**Highlights:**

**What are the main findings?**

- The transient nature of vestibular symptoms, recall bias, and clinical examination outside of the episode(s) are key challenges to accurate diagnosis.
- Selecting appropriate bedside testing is essential. While all patients with transient vestibular symptoms should undergo positional testing, HINTS+ should be avoided if no nystagmus is seen or if symptoms have already stopped.

**What are the implications of the main findings?**

- Structured history-taking and targeted ocular motor examination help in narrowing down the differential diagnosis of transient vestibular symptoms.
- The key priority is distinction between vertebrobasilar TIA, vestibular migraine, and cardiac arrhythmia.

**Abstract:**

**Background:** As many as two-thirds of acutely dizzy patients presenting to the emergency department report intermittent symptoms only, consistent with a transient acute vestibular syndrome (single episode lasting < 24 h) or an episodic vestibular syndrome (recurrent episodes, each lasting < 24 h). A broad differential diagnosis, presentation outside the episode(s), and difficulties in describing the symptoms make the diagnostic workup of transient vestibular symptoms (TVS) challenging. **Methods:** This critical review provides an overview on the management of TVS, focusing on structured history-taking and bedside testing, but also reviewing the role of neuroimaging and eye movement recordings. **Results:** Asking about timing and triggers helps clarify the temporal evolution of symptoms (transient vs. persistent) and the circumstances of occurrence (spontaneous vs. triggered). Such structured history-taking narrows down the differential diagnosis and ensures appropriate bedside tests. On clinical examination, identifying focal neurologic signs, (subtle) ocular motor findings, and gait imbalance is essential. While positional testing should be applied in all patients with TVS, HINTS+ has not been validated in those patients without nystagmus or with transient (already resolved) symptoms. For neuroimaging, MRI with diffusion-weighted imaging is preferred, but early false-negative findings in up to 20% (to even 50% for small brainstem strokes) of cases must be considered. Quantitative eye movement recordings may be especially valuable in rural areas, supporting telemedicine consults. **Conclusions:** Special emphasis should be put on distinguishing vertebrobasilar transient ischemic attack from vestibular migraine and cardiac arrhythmia. In the case of triggered TVS, immediate and sustained response to liberation maneuvers strongly supports benign paroxysmal positional vertigo over vestibular migraine or structural central positional nystagmus. Treatment strategies in TVS strongly depend on the underlying cause, ranging from hyperacute stroke treatment to migraine prophylaxis to liberation maneuvers.

## 1. Introduction

Acute-onset vertigo, dizziness, or gait imbalance represent frequent leading symptoms in patients seen in the emergency department (ED) setting, making up 2.1 to 7.1% of all ED consults [1,2,3,4,5]. This translates into more than 4 million visits to US EDs (and between 50 and 100 million consults worldwide) annually [3], generating costs of more than 10 billion US dollars per year [6]. About half of those acutely dizzy or ataxic patients suffer from general medical conditions, whereas over 10% are diagnosed with neurological disorders [3]. Eventually, 3.0–5.5% of all acutely dizzy patients will receive a diagnosis of vertebrobasilar ischemia [3,7,8]. In a large stroke surveillance study, 2% of all acutely dizzy cases had imaging-confirmed stroke, whereas 1% were diagnosed with vertebrobasilar transient ischemic attacks (TIAs) [8]. Between 1/3 and 2/3 of acutely dizzy patients report intermittent symptoms only, which may be described as transient acute vestibular syndrome (single episode lasting < 24 h) or as episodic vestibular syndrome, with one or several episodes usually lasting < 24 h [9,10].

The approach to the acutely dizzy patient critically depends on the temporal evolution of presenting symptoms and findings identified. Potential pitfalls in the assessment of the acutely dizzy patient include overreliance on the quality of symptoms and (absent) findings on (CT-based) brain imaging, applying the wrong diagnostic tests, and neglecting stroke in the young and those without focal neurologic symptoms (see [11] for review). The diagnostic workup for those patients with acute and persistent vertigo, dizziness, or gait imbalance meeting diagnostic criteria for an acute vestibular syndrome (AVS) is facilitated by the fact that symptoms are ongoing, providing the opportunity to detect subtle ocular motor signs (see [12,13] for reviews). Transient symptoms, however, represent a far greater diagnostic challenge. Limitations include an absence of clinical findings when presenting to the ED, recall bias of symptoms that have already ceased, and low impact of brain imaging in this setting.

Transient vestibular symptoms (TVS) may be a precursor of vertebrobasilar stroke, reaching as high as 12% for the preceding three months in a prospective study on vertebrobasilar stroke patients [14]. Likewise, prodromal audiovestibular symptoms may be observed in a substantial fraction of strokes involving the territory of the anterior inferior cerebellar artery (AICA), as reported in two case series (with 7.4% [15] and 31% [16], respectively). While such studies are prone to selection bias and are not representative for all dizzy patients presenting to the ED, they emphasize the need for appropriate diagnostic workup. With regard to initiating secondary preventive measures, identifying those cases early on is critical, with the aim of reducing long-term stroke-related disability. For small (lacunar) vertebrobasilar strokes, presenting symptoms and signs may be transient (i.e., lasting < 24 h) despite the confirmation of acute stroke on MR-imaging, whereas in other cases with persistent vestibular symptoms early (i.e., obtained within the first 24–48 h) MRI, including diffusion-weighted imaging (DWI), may be false-negative in up to 50% of cases [17,18]. This underlines the urgency of a thorough evaluation of the patient with acute TVS in the ED setting. However, the broad spectrum of potential underlying diagnoses—spanning almost all specialties—constitutes a further challenge.

This critical review provides an overview on the management of the patient with acute transient vertigo, dizziness, or imbalance, focusing on the diagnostic workup of selecting suitable bedside testing and structured history-taking, but also reviewing the impact (and limitations) of quantitative testing.

## 2. History-Taking in Patients with Transient Vestibular Symptoms

**Figure 1:**
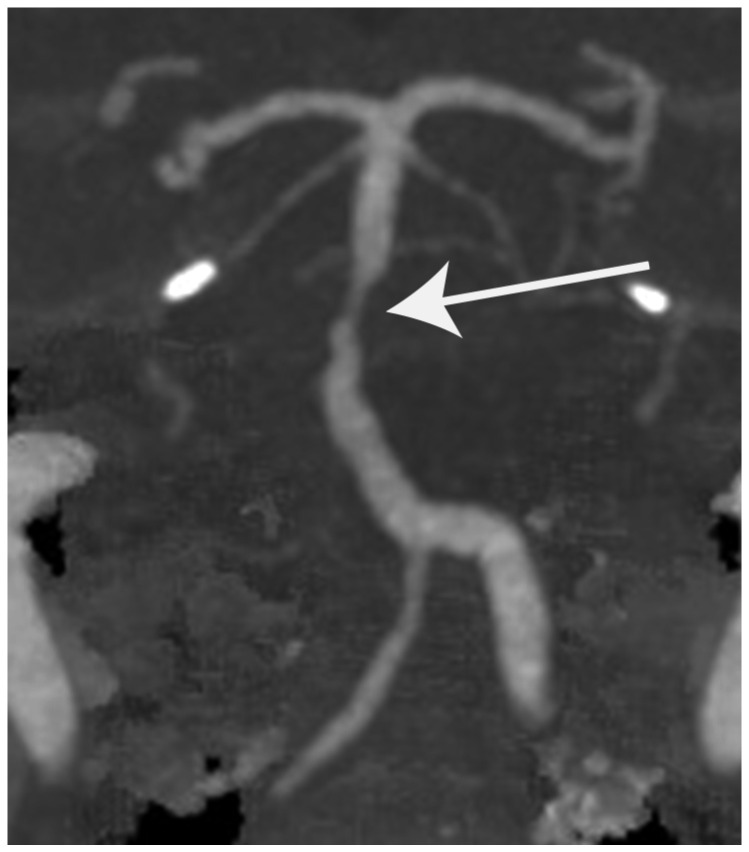
Illustrative case of a 78-year-old patient presenting with a first episode of transient vertigo and dysarthria for about 20 min. On CT-angiography a significant (i.e., >50%) stenosis of the basilar artery was detected (indicated by a white arrow). Under conservative treatment the patient continued to report recurrent episodes of transient vestibular symptoms, and thus he was referred to interventional neuroradiology for further diagnostics and treatment (courtesy of MR-image: Radiology, Cantonal Hospital of Baden, Baden).

When approaching the acutely dizzy patient in the ED, focused history-taking is key for selecting suitable bedside diagnostic tests. In line with the previously promoted TiTrATE approach [19], it is important to explore timing and the presence/absence of triggers. A principal distinction is made between spontaneous transient vestibular symptoms (sTVS) and those that were triggered (tTVS). Together with a pathophysiological distinction between central-vestibular causes, peripheral-vestibular causes, and non-vestibular causes, the differential diagnosis can be narrowed down substantially (see Table 1). Changes in head position, (fast) head movements, hyperventilation, but also moving visual surroundings or specific locations (such as crowded places, narrow hallways) constitute important triggers for specific diagnoses, whereas changes in head position relative to gravity is a key element in benign paroxysmal positional vertigo (BPPV) [20], representing the most frequent peripheral-vestibular cause of triggered episodic vestibular syndrome (EVS), and both central structural or metabolic-toxic causes and general medicine causes such as orthostatic hypotension may present with positional vertigo or dizziness also. This includes central positional vertigo due to vertebrobasilar stroke, tumors in vicinity to the fourth ventricle, but also position-dependent symptoms of vestibular migraine (VM) episodes [21]. Importantly, some disorders may present either with sTVS or tTVS, further complicating the diagnostic approach and differential diagnosis. VM is probably the best example for such versatile clinical presentation, demonstrating either spontaneous or position-dependent occurrence of symptoms, but also vertebrobasilar stroke/TIA may be position-dependent. For many other differential diagnoses, however, absence of triggers is a characteristic finding, including those with Menière’s disease and cardiac arrhythmia. Edlow and Bellolio proposed an algorithm for approaching the patient with episodic dizziness that is asymptomatic and has a normal neurologic exam at the time of evaluation (see Figure 1 in their publication), emphasizing the search for clinical factors making TIA, cardiac cause or vestibular migraine more likely [22]. Likewise, reviewing the duration of symptoms and the number of dizzy episodes noted provide additional valuable information. Importantly, acute-onset TVS may be the first episode of a recurrent, episodic vestibular disorder such as VM or Menière’s disease.

## 3. Bedside Diagnostic Testing and Algorithms in Patients with Transient Vestibular Symptoms

The selection of suitable bedside tests critically depends on the information retrieved in the patient’s history. Whereas symptoms may be ongoing, but eventually transient in nature (i.e., lasting < 24 h) in some dizzy patients, they may have stopped already in others when presenting to the ED. This has a significant impact on the management of these patients.

Importantly, the HINTS+ (head impulse, nystagmus, test of skew, new-onset unilateral hearing loss [23]) testing algorithm is validated only for patients with acute-onset, persistent vertigo/dizziness or gait imbalance with accompanying spontaneous or gaze-evoked nystagmus [11]. Other algorithms such as STANDING [24], however, are more versatile and may be applied also in patients with transient (positional) vertigo, dizziness or gait imbalance that has already been resolved at the time of evaluation (see Table 2). Thus, the diagnostic accuracy of bedside tests applied critically depends on the context. For example, applying HINTS+ to acutely dizzy patients without nystagmus bears the risk to lower the specificity to detect central (ischemic) causes, as HINTS+ will show a central pattern in those cases (head-impulse test bilaterally normal, no skew deviation, no gaze-evoked nystagmus, no hearing loss). This may result in unnecessary diagnostic testing and referral to a stroke unit.

In all patients with TVS (either ongoing or resolved), a focal neurologic examination and a structured eye exam including an evaluation of gaze stability (looking for spontaneous nystagmus, gaze-evoked nystagmus, saccadic intrusions and ocular lateral deviation [28]) with fixation preserved and removed, saccadic eye movements (with regard to latency, velocity and metrics) and pursuit eye movements, and the integrity of the vestibulo-ocular reflex is recommended. If symptoms are ongoing and presence of a (spontaneous or gaze-evoked) nystagmus is confirmed, the diagnostic accuracy of the HINTS+ algorithm is high (exceeding 95% for sensitivity when applied by at least moderately trained physicians [29]). The pattern of spontaneous nystagmus should be assessed in these cases as well. Albeit found in a minority of patients only, (isolated) vertical and/or torsional spontaneous nystagmus is highly predictive for a central cause when present [30]. In addition, a graded rating of any truncal and gait instability (GTI) should be retrieved in the acutely dizzy patient. This is because an inability to stand or sit unassisted is highly predictive for a central cause [31].

If symptoms have already stopped at the time of examination, searching for a triggered nystagmus by means of head-shaking, skull vibration, hyperventilation and positional testing is recommended (see [32] for a discussion of the value of triggered nystagmus patterns in acutely dizzy patients). Specifically, due to the high prevalence of BPPV in the population and excellent treatment response, every patient with TVS should receive positional testing by applying the Dix–Hallpike test (assessing the posterior (and anterior) canals) and the supine-roll test (assessing the lateral canals). This includes also those patients that denied triggers on history-taking. Importantly, patients presenting with a first episode of VM may report positional (transient) vertigo or dizziness only and may demonstrate a positional nystagmus as well [33]. In comparison to BPPV, positional nystagmus in VM is typically less intense (lower amplitude, lower frequency), persistent and does not response to liberatory maneuvers. Noteworthy, the positional nystagmus patterns observed may be overlapping. Specifically, in most cases of VM a horizontal (geotropic or apogeotropic) persistent nystagmus is seen on supine-roll testing (resembling lateral-canal BPPV), but also purely torsional nystagmus may be observed in Dix–Hallpike testing (potentially being misinterpreted as posterior-canal BPPV).

If the bedside examination and diagnostic testing do not demonstrate any signs suggesting a peripheral- or central-vestibular disorder, the diagnostic approach should be shifted towards non-neurological (non-vestibular), general medical causes (see also the recent GRACE3 guideline on how to approach the acutely dizzy patient [34]). The broad range of non-neurological causes in the acutely dizzy patient has been reviewed previously in detail elsewhere [12]. In brief, transient vertigo, dizziness or gait imbalance of non-vestibular origin may be related to cardiac arrhythmia, electrolyte disturbances (including hyponatremia), hypo-/hyperglycemia, anemia and other general medical conditions (see also Table 1).

At first presentation, the differential diagnosis is very broad, including both monophasic disorders with low risk of recurrence and recurrent disorders. Thus, acute TVS may represent the first episode of an EVS such as VM, Menière’s disease or recurrent cardiac arrhythmia [35]. After the first episode, diagnostic criteria for these entities are often not met, allowing a provisional diagnosis only if at all [35]. This is especially true for VM, requiring a minimum of five vestibular episodes to confirm the diagnosis according to current ICVD diagnostic criteria [36]. Recurrent TVS in these patients may therefore over-proportionally bind ED resources including repeated acute brain imaging and hyperacute stroke treatment, despite the much more benign nature of the underlying disease. Thus, whenever assessing the patient with TVS, targeted history should include asking for previous, similar episodes and the differential diagnosis should include recurrent (episodic) vestibular disorders as well.

## 4. Quantitative (Vestibular) Testing in Patients with Transient Vestibular Symptoms

Decision to order quantitative testing in transiently dizzy patients should be guided by reported symptoms, the clinical examination, and availability. Brain imaging (preferentially MRI-based, including MR-angiography) should be retrieved in those with a suspected central cause, especially if a vertebrobasilar TIA is considered (see Figure 1 for an illustrative case), but also in those patients with a migraine headache history and changes in clinical presentation (e.g., a first episode of possible VM). CT-based imaging may be considered if focal neurologic signs or subtle ocular motor signs point to a central origin and onset is hyperacute. Thus, CT including CT-angiography may provide useful in identifying those cases with basilar artery stenosis, dissection or even occlusion, that may require immediate systemic or endovascular treatment. Importantly, CT-based imaging has a sensitivity as low as 14.3% (for CTA) and 21.4% (for non-contrast CT), respectively, to detect acute vertebrobasilar ischemia [17]. Furthermore, also CT-perfusion based imaging is limited for infratentorial strokes (especially brainstem strokes due to the small volume and occurring artifacts due to surrounding structures). Limitations of brain imaging in patients with TVS is further discussed here [37].

In those patients that reported new ear symptoms including new-onset unilateral hearing loss, tinnitus or aural fullness, a pure-tone audiometry (PTA) should be carried out. If no access to PTA is available, hearing may be assessed using a smartphone app such as the hearWHO app [38]. This is especially valuable in patients with a first episode of combined audiovestibular symptoms to quantify the extent and distribution of hearing loss. While a (transient) low-frequency hearing loss points to a first episode of Menière’s disease (see [39] for diagnostic criteria), VB-TIAs involving the AICA territory (and thus the central auditory pathways) and isolated labyrinthine infarction are in the differential diagnosis.

Quantitative vestibular testing may be considered in those acutely dizzy patients that demonstrate subtle ocular motor signs on bedside examination despite cessation of symptoms. Especially in remote places, implementation of telemedicine approaches and thus gaining access to expert knowledge may provide very valuable. This may include the application of video-HINTS (if a spontaneous or gaze-evoked nystagmus is seen) as described by Korda and colleagues [40], thus assessing both eccentric gaze holding, vertical ocular alignment and the integrity of the vestibulo-ocular reflex. Alternatively (or additionally), video-oculography (VOG) may be obtained to quantify gaze stability, saccadic eye movements, pursuit and triggered nystagmus (including positional testing). With new, emerging technologies providing VOG on portable devices including smartphones, access to such advanced diagnostic approaches will be facilitated, which may improve diagnostic accuracy and eventually also outcome.

In those patients presenting to the ED with suspected non-neurological causes, established diagnostic pathways including blood workup reliably allow for the detection of anemia, electrolyte disturbances or glucose metabolism disorders. In addition, assessing blood pressure and heart frequency lying and standing may provide useful in those with suspected orthostatic hypotension or postural orthostatic tachycardia syndrome (POTS), whereas electrocardiography (ECG) is key to detect possible heart rhythm disorders.

## 5. Key Central-Vestibular Disorders Presenting with Transient Signs and Symptoms

A broad range of central disorders may present with TVS. Here we review the most frequent and the most dangerous ones.

### 5.1. Vertebrobasilar Transient Neurological Attacks/TIA

Amongst central-vestibular disorders presenting with transient signs and symptoms, vertebrobasilar TIA is the most important diagnosis not to miss because of the risk of recurrence and persistent disability. While the National Institute of Neurological Disorders and Stroke criteria do not define transient isolated dizziness/vertigo as a focal symptom of TIAs [41] and thus has been referred to as transient neurological attack (TNA), diagnostic criteria for transient vascular vertigo have been recently published [42]. Importantly, the combination of brief symptom duration (usually minutes, sometimes up to few hours [14,43,44]), focal neurologic signs, absence of MRI-DWI positive lesions, increased risk for vascular events (such as an ABCD2-score of ≥4, untreated atrial fibrillation), and significant (>50%) narrowing of an artery of the vertebrobasilar system makes a VB-TIA likely (with the label “probable transient vascular vertigo/dizziness” according to the diagnostic criteria). However, obvious focal neurologic signs may be missing in VB-TIA or may be non-localizing such as in the case of dysarthria. This makes the diagnostic workup even more challenging. The risk of misdiagnosis for cerebrovascular events is much higher when presenting neurologic complaints are mild, nonspecific (dizziness vs. motor symptoms), or transient (range 24–60%) as emphasized in a meta-analysis [45]. Edlow and Bellolio estimated, that about 15% of all posterior circulation TIAs present with isolated dizziness [22]. This translates to about 10,000 patients with VB-TIA presenting with isolated dizziness in the US every year. These numbers are in line with the 15.5% of isolated VB-TIAs in a Brazilian cohort [46], but were higher than those observed in a multicenter Canadian study including patients presenting to the ED with TIA, where 4.2% (484/11,507) of patients suffered from isolated dizziness [47].

#### 5.1.1. Incidence of Posterior Circulation Stroke/TIA in Acutely Dizzy Patients

Several studies have reported on the incidence of stroke/TIA amongst acutely dizzy patients. In a prospective, single-center observational study focusing on patients with acute transient vestibular syndrome (ATVS, i.e., lasting < 24 h), MRI-DWI and cerebellar hypoperfusion on perfusion-weighted imaging were used to confirm stroke or vertebrobasilar TIA. Cases with BPPV, general medicine causes or known neurological disorders were excluded. Importantly, HINTS+ could not be applied in 73% of patients since symptoms had already ceased and initial MRI-DWI was false-negative in 43% of confirmed strokes [43]. Overall, they found a prevalence of stroke of 15% and one of TIA of 12% in their cohort. When focusing on the asymptomatic ATVS cases, numbers for stroke (17%) and TIA (14%) were even higher. Furthermore, both craniocervical pain and focal neurological signs/symptoms were overrepresented in the stroke cases compared to the non-stroke ATVS cases. In this study, MR-based perfusion-weighted imaging (PWI) demonstrated cerebellar hypoperfusion without DWI lesions (on repeated MRI) in 10/86 cases with transient AVS [43]. Overall, 8 out of those 10 patients had a focal stenosis or hypoplasia of the corresponding vertebral artery. Thus, hypoperfusion on MRI-PWI without DWI lesions could be a suitable biomarker to support a central, transient ischemic cause of the patient’s acute vertigo. Noteworthy, access to MRI-PWI may be not be readily available, limiting the value of this approach.

One limitation of this study is that VM was not systematically searched for. In another study, using data from the EMVERT trial from a large German tertiary hospital, 15.7% of acutely (transiently) dizzy patients (symptom duration > 10 min) suffered from stroke and 8.6% were diagnosed with TIA [48]. In this study also vestibular migraine cases (8.2%) and migraine-like acute transient vestibular syndromes (4.2%), narrowing this gap. 

In another study reporting on acutely dizzy patients presenting to the ED with episodic vestibular symptoms (first episode or repeated episodes), cerebrovascular case numbers were somewhat lower, most likely related to the broader inclusion criteria. Specifically, 10% of cases were diagnosed with TIA and 2% with a stroke [10]. From those patients that received a diagnosis of VB-TIA in the ED, 14.3% suffered a stroke on follow up (up to 90 days after the index event) in this cohort. 

#### 5.1.2. Transient Vestibular Symptoms as a Warning Sign of Stroke/TIA

TVS may be a warning sign of stroke. In a retrospective, 4-year follow-up study, patients that had been hospitalized because of acute isolated vertigo of any cause (except for central vertigo) according to the ICD-9-CM code had a three-times-higher risk of stroke than the general population [49]. In addition, vertigo patients with ≥3 vascular risk factors had a 5.5-fold higher risk of stroke than those without vascular risk factors. Notably, in this study, no information on the cause of vertigo or the symptom duration was provided; however, focusing of hospitalized patients makes certain diagnoses such as BPPV or orthostatic hypotension less likely.

Several studies have retrieved the frequency of previous vestibular symptoms in patients presenting with posterior circulation stroke (PCS) or TIA. In a prospective, population-based incidence study, 21.5% (59/275) of PCS patients reported subtle transient neurological symptoms in the 90 days preceding their stroke, most frequently vertigo (12%), being isolated in 23/275 patients and non-isolated in 10/275 cases [44]. Likewise, in a prospective multicenter observational study including patients with PCS, the rate of TVS (vertigo, dizziness or imbalance) within the previous 3 months was 12% (55/447), with one third of events having occurred within one week of the stroke [14]. Transient imbalance was reported in 40% of cases, being present in isolation in only 4% of cases. Noteworthy, those 16% of patients with >10 episodes of TVS were diagnosed with atrial fibrillation more frequently. In a prospective study including 103 patients presenting with acute transient vertigo or dizziness diagnosed as VB-TIA, secondary prophylaxis reduced the frequency of events by 93.2% [95% CI: 88.34–98.06; number needed to treat: 1] during a median follow-up of 12 months (range: 2–36 months). Only seven (6.8%) patients experienced a new attack while on medication. A broad and comprehensive evaluation was performed, including 14-day ECG-Holter monitoring. The potential value of initial stroke workup and initiation of secondary preventive treatment is underlined by these numbers. However, the challenge remains to identify the subset of patients with TVS due to VB-TIA and in such studies looking back at preceding events, their underlying cause was not investigated and thus is subject to interpretation. 

In another, retrospective single-center study on patients presenting with isolated transient vertigo, 48/339 (14.2%) initially received a diagnosis of probable or definitive cerebrovascular vertigo [50]. On follow-up (range 3 days to 7.7 years), 41/339 suffered from stroke or TIA, with 26/41 cases affecting the posterior circulation. Noteworthy, one or more vascular risk factors was present in 85.5% of patients in this cohort, indicating a potential selection bias. Surprisingly, the initial diagnosis by the stroke neurologist was not correlated with the future risk for stroke, pointing to the limitations in diagnostic accuracy in this setting.

### 5.2. TVS Related to Vestibular Migraine (VM)

An important differential diagnosis of VB-TIA is a first episode of VM. Negative brain imaging, presence of focal neurologic signs and subtle ocular motor findings are all overlapping with VB-TIA. Amongst a cohort of acutely dizzy patients presenting to the ED with episodic vestibular symptoms, a diagnosis of VM was made in 31/533 (6%), whereas vertebrobasilar TIA was found in 55/533 (10%) and diagnosis remained unclear in 239/533 (44.8%) [10].Younger age, female gender, a history of migraine headaches and accompanying migraineous features (including migraine headache, photo-/phonophobia) and lack of vascular risk factors make a diagnosis of VM more likely. Due to hyperacute presentation, disabling symptoms and lacking biomarkers for VM, these patients may receive hyperacute stroke treatment before the diagnosis is confirmed (see Supplementary Video S1 for a case with positional vertigo and suspected VM). When presenting with disabling neurologic symptoms and suspected stroke, hyperacute treatment should not be delayed in case a stroke mimic cannot be excluded according to current guidelines [51]. Overall, intravenous thrombolysis in stroke mimics is considered safe, with very low rates of symptomatic intracranial hemorrhage (0.5%) or orolingual edema (0.3%) in a meta-analysis [52].

### 5.3. TVS Related to Drug or Alcohol Intoxication

Drug intoxication may result in TVS, typically lasting hours. Antiseizure medication (phenytoin, carbamazepine, oxcarbazepine, lacosamide and others), and neuroleptics are amongst the most frequent causes of metabolic-toxic transient vestibular symptoms. Drug interactions (e.g., reduced elimination due to competing mechanisms), acute renal or liver failure or errors in intake are potential causes of intoxication. Based on a review of current medication, patient history and clinical examination, the diagnosis can be readily made in most cases. Subtle ocular motor findings pointing to either peripheral- or central-vestibular impairment (including spontaneous nystagmus, gaze-evoked nystagmus and downbeat nystagmus) may be observed, being transient in nature and reversible after drug cessation. Noteworthy, biomarkers (drug blood level concentrations) are available only with delays of several days for most substances, thus not contributing to acute decision making. Alcohol intoxication demonstrating acute cerebellar loss of function may initially be misinterpreted as vertebrobasilar stroke/TIA, However, a positive history of alcohol consumption, confirmed high blood alcohol concentrations and lack of localizing neurologic signs usually allow for the right diagnosis.

### 5.4. Other Central Causes Including Epileptic Vertigo and Episodic Ataxia

Epileptic vertigo was found in 8.5% of epileptic seizures in a systematic review, being very brief (<30 s) in most cases [53]. It is rarely (<1%) observed in isolation and accompanying findings usually allow for the correct diagnosis of an epileptic seizure reliably. Noteworthy, due to different cortical areas triggering epileptic vertigo and epileptic nystagmus, combined occurrence is rarely seen. However, a seizure may be acute and symptomatic in the setting of an acute stroke; thus, the differential diagnosis needs to be kept broad, including vestibular paroxysmia (see [54] for diagnostic criteria). Hereditary cerebellar ataxia may present with episodic symptoms. This is true both for spinocerebellar ataxia 27B during early disease stages [55] and for the episodic ataxias (see [56] for a review). Most patients will demonstrate persistent (subtle) ocular motor and vestibular symptoms pointing to an underlying persistent cerebellar ataxia.

### 5.5. Triggered TVS of Central Origin

Central positional vertigo and nystagmus may be observed both in cases with structural lesions around the fourth ventricle (including demyelinating lesions, strokes and tumors), but also in cases with VM or vertebrobasilar TIA (see Supplementary Video S2). Thus, in patients with position-dependent TVS the pattern of positional nystagmus should be thoroughly analyzed. Hints to a central origin include a positional nystagmus not matching the plane of the stimulated semicircular canal, lack of sustained treatment response, and positional vomiting. Most frequently, central positional nystagmus is apogeotropic horizontal on supine roll [21], a pattern infrequently seen in BPPV.

In cases with TVS being triggered by head rotation or head tilt, also a possible diagnosis of vertebral artery compression syndrome (VACS) should be considered. Hypoplasia or stenosis of one vertebral artery in combination with compression/occlusion of the contralateral (dominant) vertebral artery—usually at the level of the atlantoaxial junction—is the presumed underlying pathomechanism. Clinically, upon head position change various nystagmus patterns may be observed, including downbeat nystagmus and gaze-evoked nystagmus [57,58]. For confirmation of diagnosis usually digital subtraction angiography is needed.

In cases of head position-dependent vertigo and (pre-)syncope, a hypersensitive carotid sinus should be considered as well. Episodes are usually triggered by head rotations or by wearing a tight tie. Diagnosis is usually confirmed by demonstrating bradycardia upon carotid sinus massage (after assessing that there are no atherosclerotic changes in the carotid arteries), a procedure that is usually done by an experienced cardiologist.

## 6. Peripheral-Vestibular Disorders Presenting with Transient Signs and Symptoms

Amongst peripheral-vestibular disorders that may present with TVS, BPPV is the most common cause (with an annual incidence of about 0.6% [59]). For presenting symptoms of short-lasting TVS (duration < 60 s) being triggered by head position changes relative to gravity, BPPV is by far the most likely diagnosis and should be systematically searched for. Thus, provocation maneuvers (Hallpike–Dix maneuver and supine-roll maneuver) should be always applied if symptoms have stopped. Importantly, positional vertigo and nystagmus may also be seen in VM. Thus, a BPPV diagnosis should be made only in light of confirmed immediate and lasting treatment response to liberation maneuvers. In a retrospective single-center study on transient isolated vertigo or dizziness, 17.9% of cases received a diagnosis of posterior-canal BPPV [50], whereas in another study 108/194 (55.7%) cases with ATVS were diagnosed with BPPV [43]. Interestingly, in this study vertigo triggered by a change in head position was associated with lower stroke risk. This reflects the distribution of diagnoses related to transient positional vertigo, with a minority of 11–12% only receiving a diagnosis of central positional vertigo [60,61].

A diagnosis of potential Menière’s disease is possible in those cases with transient combined audiovestibular symptoms, although the diagnosis should not be made outside specialist centers. A transient peripheral-vestibular pattern (lasting minutes to hours) at the bedside with either irritative nystagmus or loss-of-function nystagmus and aural symptoms are characteristic features (see [39] for diagnostic criteria). If the episode has already stopped when presenting to the ED, history-taking is key. Nonetheless, audiovestibular testing in these cases may be helpful to detect residual, subtle abnormalities. MR-Imaging including delayed contrast-enhanced sequences of the inner ear, demonstrating endolymphatic hydrops may support the diagnosis, but is often not readily available. Likewise, vestibular paroxysmia or other, less frequent causes of spontaneous, short-lasting vestibular symptoms (see Table 1) should be considered.

## 7. Non-Vestibular Disorders Presenting with Transient Vertigo, Dizziness or Imbalance

A broad range of non-vestibular disorders may result in acute, but transient imbalance, vertigo or dizziness, for an in-depth review of the differential diagnosis see [12]. Here we will focus on the most common and most dangerous non-vestibular disorders leading to transient spontaneous or triggered vertigo, dizziness or gait imbalance.

In a cross-sectional retrospective study of data from the large US National Hospital Ambulatory Medical Care Survey (NHAMC) database, general medical diagnoses (49.2%) were more commonly identified than otovestibular diagnoses (32.9%) amongst 9472 acutely dizzy patients presenting to the ED [3]. The largest subgroup within general medical diagnoses constituted cardiovascular diagnoses, making up 21.1% when aggregated with peripheral vascular disorders (no separate numbers provided). Importantly, no distinction between transient and persistent dizziness was made in this study. We can only speculate about the fraction of dizzy cases with transient symptoms (i.e., with <24 h duration), but amongst specific general medical diagnoses listed in this study, some will be most likely transient in nature. Focusing on those with spontaneous occurrence, this includes acute dizziness related to fluid and electrolyte disorders (5.6%), cardiac arrhythmia (3.2%), hypoglycemia (1.4%), angina (0.9%), panic disorders (0.5%), and carbon monoxide poisoning (0.2%). Likewise, vasovagal syncope (6.6%), anemia (1.6%), and orthostatic hypotension (0.6%) were the most frequent general medical disorders leading to triggered and likely transient vertigo or dizziness identified in this study. In another study, 3/194 cases with ATVS were diagnosed with cardiac arrhythmia, 7/194 with orthostatic hypotension [43]. As mentioned in the diagnostic approach section of this review, many of these disorders will be readily identified due to standard operational procedures in the ED, including ECG recordings, blood workup and structured history-taking. Special focus should be put to identify potentially dangerous conditions such as cardiac arrhythmia and fluid/electrolyte disturbances.

## 8. Limitations and Outlook

The diagnostic algorithms for those patients with TVS that have already resolved need further refinement. Therefore, prospective observational studies are urgently needed to allow for a better characterization of the spectrum of clinical presentations and parameters predicting outcome. The UTRAVERA study protocol (clinicaltrials.gov: NCT07421973) offers such an outlook focusing on transient neurological symptoms in acutely dizzy patients. However, this multicenter international study is far from being completed at this time.

## 9. Conclusions

Structured patient history-taking and targeted bedside examination are key in the patient with TVS. Overreliance on CT- or MRI-based acute imaging should be avoided and bedside testing needs to be selected based on reported symptoms and triggers at the time of examination (ongoing vs. ceased) and neurologic findings including assessing for subtle ocular motor abnormalities. While in the setting of ongoing vestibular symptoms absence of focal neurologic signs and subtle ocular motor signs points to a non-vestibular (general medical) condition, narrowing the differential diagnosis is much more challenging in those that present only after symptoms have already stopped.

## Figures and Tables

**Table 1:** Transient vestibular symptoms—distinction by duration of single episodes.

	Seconds	Minutes	Minutes to Hours
Spontaneous	Cardiac arrhythmia Vestibular paroxysmia Epileptic vertigo Other, presumably rare causes including paroxysmal brainstem attacks in MS due to demyelination, ocular neuromyotonia and superior oblique myokymia	Panic attacks Hypoglycemia Cardiac arrhythmia	Vertebrobasilar TIA Vestibular migraine Alcohol intoxication Drug intoxication Carbon monoxide poisoning Hyponatremia, hypomagnesemia Episodic ataxias and SCA27B Menière’s disease Hypertensive encephalopathy
Triggered (head position-dependent, situational)	BPPV CPV	Vestibular migraine Orthostatic hypotension POTS Vertebral artery compression syndrome Hypersensitive carotid sinus Subclavian steel syndrome	Somatoform dizziness (situational triggering)

Abbreviations: BPPV = benign paroxysmal positional vertigo; CPV = central positional vertigo; POTS = postural orthostatic tachycardia syndrome; SCA = spinocerebellar ataxia; TIA = transient ischemic attack.

**Table 2:** Suitable diagnostic testing in acutely dizzy patients.

	Recommended Bedside Testing	Algorithms to Consider	Recommended Quantitative Testing	Comments
Acute-onset, transient (i.e., already resolved) vertigo/dizziness	Focal neurological and neuro-otological examination Subtle ocular motor exam including gaze palsies, gaze holding, pursuit, saccades, VOR Testing for triggered nystagmus (HSN/VIN) Positional testing (Dix–Hallpike, Supine-roll test)	STANDING * Sudbury vertigo risk score [25] TriAGe+ score [26]	MRI-DWI in those with suspected central origin ECG/Holter Measuring blood pressure lying and standing Hearing test (PTA, smartphone app) Blood workup including blood count, glucose levels and electrolytes	No HINTS+ in transient cases
Acute-onset, ongoing vertigo/dizziness	Focal neurological and neuro-otological examination Subtle ocular motor exam (details see above) Examination of stance and gait	HINTS+ if SN/GEN observed * STANDING * Sudbury vertigo risk score SAV3E score [27] TriAGe+ score [26]	Video-HINTS if SN/GEN observed Hearing test (PTA, smartphone app) MRI-DWI in those with suspected central origin CT/CTA in those cases with urgent treatment decisions (IVT/EVT)	No provocation maneuvers in case of ongoing vertigo/dizziness recommended

* Preferred tests based on supporting evidence available. Abbreviations: CT = computed tomography; CTA = CT-angiography; DWI = diffusion-weighted imaging; ECG = electrocardiography; EVT = endovascular thrombectomy; GEN = gaze-evoked nystagmus; HINTS+ = head impulse, nystagmus, test of skew, new-onset unilateral hearing loss; HSN = head-shaking nystagmus, IVT = intravenous thrombolysis; MRI = magnetic resonance imaging, PTA = pure-tone audiometry; SN = spontaneous nystagmus; VIN = vibration-induced nystagmus; VOR = vestibulo-ocular reflex.

## Data Availability

The raw data supporting the two cases presented in this article will be made available by the authors on request.

## Supplementary Materials

The following supporting information can be downloaded at: https://www.mdpi.com/article/10.3390/brainsci16070754/s1, Video S1: Illustrative case presenting with acute positional vertigo and nystagmus, video legend: This 56-year-old female patient was referred to the ED with an acute-onset, positional vertigo accompanied by severe nausea and vomiting, blurred vision and gait imbalance since about 4 h for a suspected stroke. She is known for a history of episodic migraine without aura. On initial clinical examination a right-beating horizontal-torsional spontaneous nystagmus was observed, following Alexander’s law and being much worse in supine position than in upright position. No skew deviation or gaze-evoked nystagmus was seen, but gait was severely impaired (grade 3 graded truncal instability). The head-impulse test was bilaterally normal, thus HINTS were central. Due to the intense positional nausea and vomiting, intubation was necessary before emergency CT-based imaging could be performed. Imaging was normal, especially no large vessel stenosis/occlusion or hypodensities were detected. With hyperacute, clearly disabling symptoms and no contraindications for intravenous thrombolysis, treatment with alteplase was started. After extubation, about four hours later, the patient was asymptomatic, the stroke workup including delayed MRI-DWI was negative. More detailed history-taking revealed a diagnosis of migraine headaches since childhood and recurrent episodes of mild to moderate vertigo lasting hours in recent years, but never as severe as this episode. Based on this, a diagnosis of suspected vestibular migraine was made. Video S2: Another case illustrating positional (central) upbeat nystagmus, in a diabetic and hypertensive patient experiencing transient spontaneous imbalance, video legend: This 72-year-old man, who had a history of diabetes and arterial hypertension, presented to the ED with acute imbalance that had occurred spontaneously and lasted for approximately six hours. On arrival, he reported persistent symptoms rated as 7/10 on a visual analog scale. The patient arrived on foot with assistance. The patient denied any other neurological symptoms, such as visual impairment, dysarthria, dysphagia, new-onset headache, paresis, and sensory deficits. On bedside examination no obvious focal neurological signs were seen. Likewise, no subtle ocular motor findings including spontaneous nystagmus, gaze-evoked nystagmus, or head-shaking nystagmus were seen. During positional testing, the Dix–Hallpike maneuver (on either side) and the straight-head hanging position elicited upbeat positional nystagmus. Notably, the patient reported no change in the intensity of his symptoms during positional testing, maintaining the same perception of imbalance (VAS 7/10) in all tested positions. A brain MRI showed no evidence of acute ischemia, whereas magnetic resonance angiography demonstrated significant narrowing of the left V4 vertebral artery segment. After the symptomatic episode had resolved, positional testing no longer elicited nystagmus. The episode was interpreted as spontaneous acute vestibular syndrome accompanied by central positional upbeat nystagmus, most likely representing vertebrobasilar TIA. The patient remained asymptomatic for four months while taking 100 mg of aspirin daily. This case highlights the value of positional tests in the diagnosis of TSVs, even in patients who deny experiencing any triggers during the medical history-taking.

## Abbreviations

The following abbreviations are used in this manuscript:

AICA	Anterior inferior cerebellar artery
ATVS	Acute transient vestibular syndrome
AVS	Acute vestibular syndrome
BPPV	Benign paroxysmal positional vertigo
CPN	Central positional nystagmus
CT	Computed tomography
ECG	Electrocardiogram
ED	Emergency department
EVS	Episodic vestibular syndrome
EVT	Endovascular thrombectomy
GEN	Gaze-evoked nystagmus
GTI	Graded truncal instability
HINTS	Head impulse, nystagmus, test of skew
HSN	Head-shaking nystagmus
IVT	Intravenous thrombolysis
MRI-DWI	Magnetic resonance imaging with diffusion-weighted imaging
PCS	Posterior circulation stroke
PFO	Patent foramen ovale
POTS	Postural orthostatic tachycardia syndrome
PTA	Pure-tone audiogram
SCA	Spinocerebellar ataxia
SN	Spontaneous nystagmus
TIA	Transient ischemic attack
TiTrATE	Timing, triggers and targeted examination
TVS	Transient vestibular symptoms
VACS	Vertebral artery compression syndrome
VB	Vertebrobasilar
VIN	Vibration-induced nystagmus
VM	Vestibular migraine
VOG	Video-oculography
